# Supplementary material for: The locking mechanism of human TRPV6 inhibition by intracellular magnesium
Source: Nat Commun. 2025 Nov 6;16:9826. doi: 10.1038/s41467-025-65919-1 (PMC12592368; doi:10.1038/s41467-025-65919-1)
Supplement: Supplementary file 1 — Supplementary Information [file 41467_2025_65919_MOESM1_ESM.pdf]

Supplementary Information

The locking mechanism of human TRPV6 inhibition by intracellular magnesium

Arthur Neuberger[1,*], Alexey Shalygin[2,*], Irina I. Veretenenko[3,*], Yury A. Trofimov[3], Thomas Gudermann[2,4], Vladimir Chubanov[2,#], Roman G. Efremov[3,5,6,#], Alexander I. Sobolevsky[1,#]

[1] Department of Biochemistry and Molecular Biophysics, Columbia University, New York, NY 10032, USA.

[2] Walther-Straub Institute of Pharmacology and Toxicology, LMU Munich, 80336 Munich, Germany.

[3] Shemyakin-Ovchinnikov Institute of Bioorganic Chemistry, Russian Academy of Sciences, 117997 Moscow, Russia.

[4] Comprehensive Pneumology Center, German Center for Lung Research, 81377 Munich, Germany.

[5] National Research University Higher School of Economics, 101000 Moscow, Russia.

[6] Research Institute for Systems Biology and Medicine, Moscow, 117246 Moscow, Russia.

* These authors contributed equally: Arthur Neuberger, Alexey Shalygin, Irina I. Veretenenko.

[#] e-mail: vladimir.chubanov@lrz.uni-muenchen.de; efremov@nmr.ru; as4005@cumc.columbia.edu

This PDF file includes:

Supplementary Figures 1-9

Supplementary Table 1

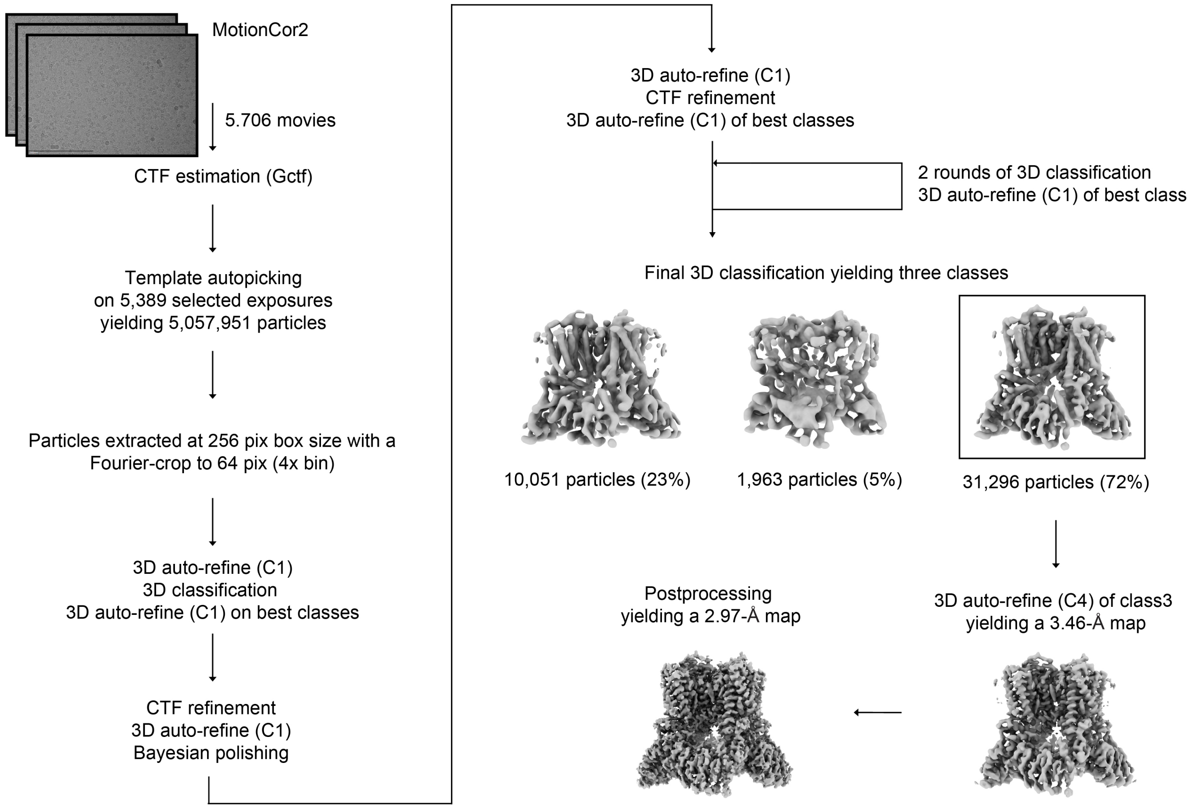

Supplementary Figure 1. 3D reconstruction workflow for hTRPV6$_{Mg}$. 3D reconstruction workflow steps are illustrated by examples of micrographs and cryo-EM densities.

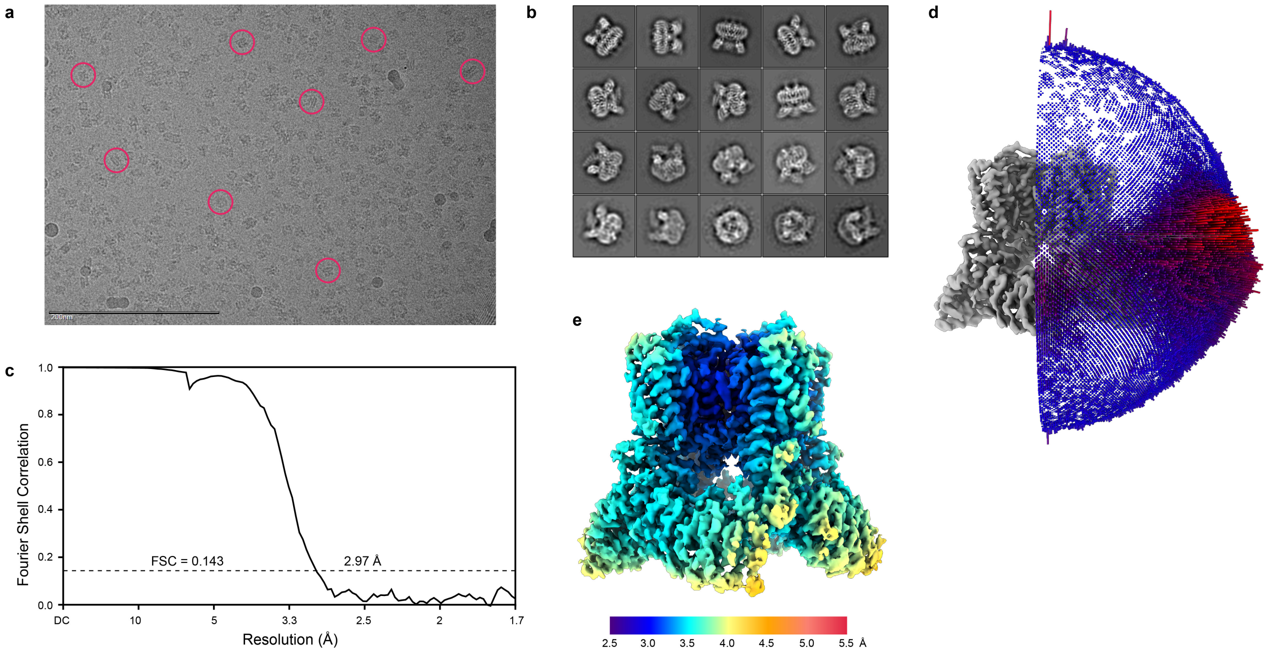

Supplementary Figure 2. Overview of cryo-EM data for hTRPV6$_{Mg}$. a, Representative micrograph with example particles circled in pink. **b**, 2D class averages. **c**, FSC curve. **d**, Euler angle distribution of particles contributing to final reconstructions of hTRPV6$_{Mg}$, with larger red cylinders representing orientations comprising more particles. **e**, Local resolution presented as coloring of the hTRPV6$_{Mg}$ cryo-EM map.

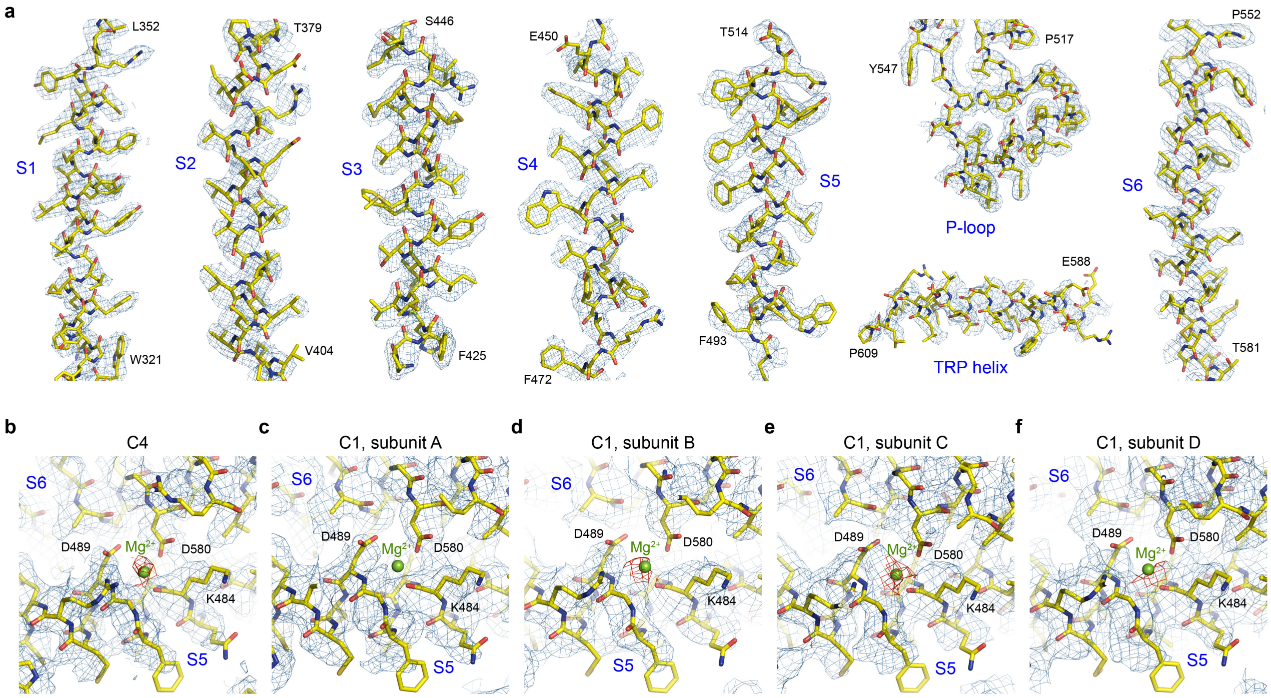

Supplementary Figure 3. Cryo-EM density for hTRPV6$_{Mg}$. a, Fragments of the TMD for hTRPV6$_{Mg}$, with the structural model shown as yellow sticks and the corresponding cryo-EM density as a blue mesh. **b**-**f**, Mg^{2+} binding sites in hTRPV6$_{Mg}$ for the 3D reconstruction obtained using C4 (**b**) or C1 (**c**-**f**) symmetry, with cryo-EM density for Mg^{2+} ions shown as red mesh.

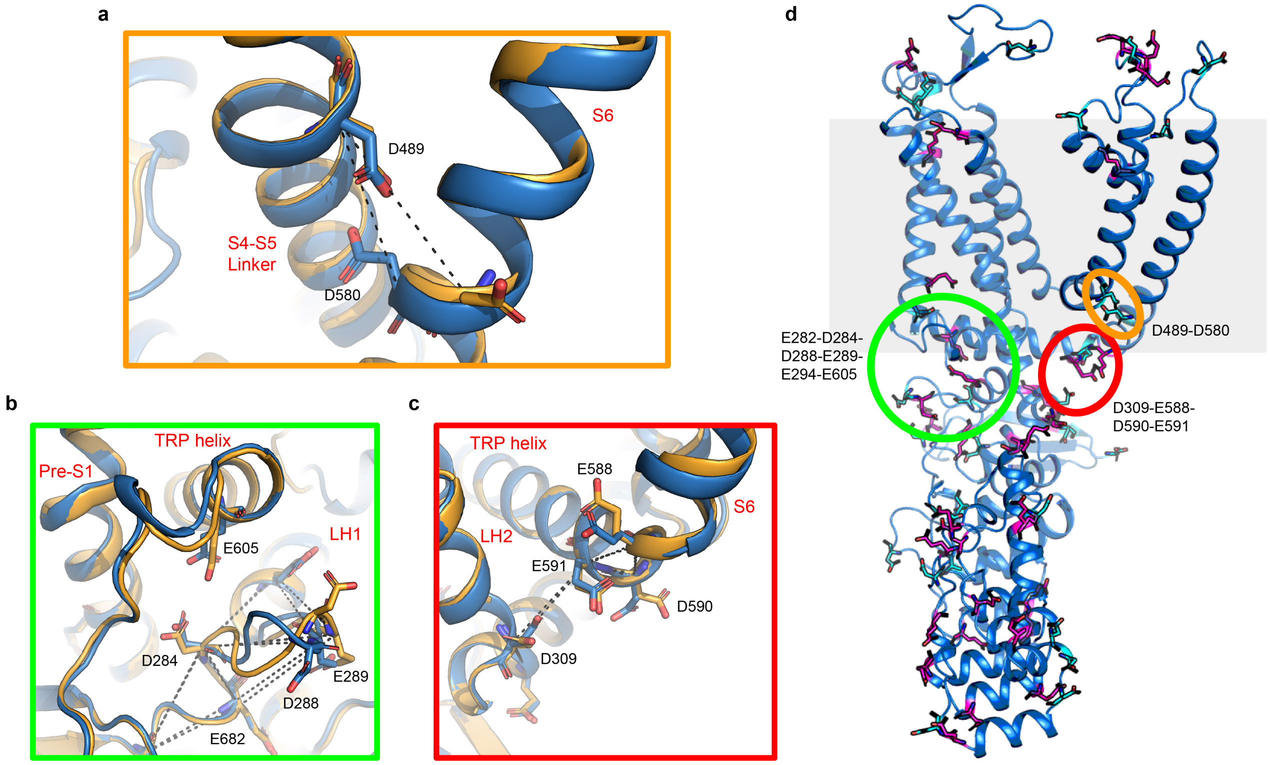

Supplementary Figure 4. Identification of potential hTRPV6 intracellular Mg^{2+} binding sites. **a-c**, Identified potential Mg^{2+} binding sites in $hTRPV6_{Mg}$ (blue) and $hTRPV6_{Open}$ (orange) on the intracellular side of the TMD: (**a**) the D489-D580 site between S5 and S6, (**b**) the E282-D284-D288-E289-E294-E605 site between the LH1 helix and the C-terminus of the TRP helix and (**c**) the D309-E588-D590-E591 site between the S6-TRP helix kink and the LH2 helix. **d**, Mapping of these sites on a single $hTRPV6_{Mg}$ subunit. All acidic residues are shown as cyan (D) and magenta (E) sticks, the identified sites are marked with colored ellipses, and the TMD region is highlighted with a grey rectangle.

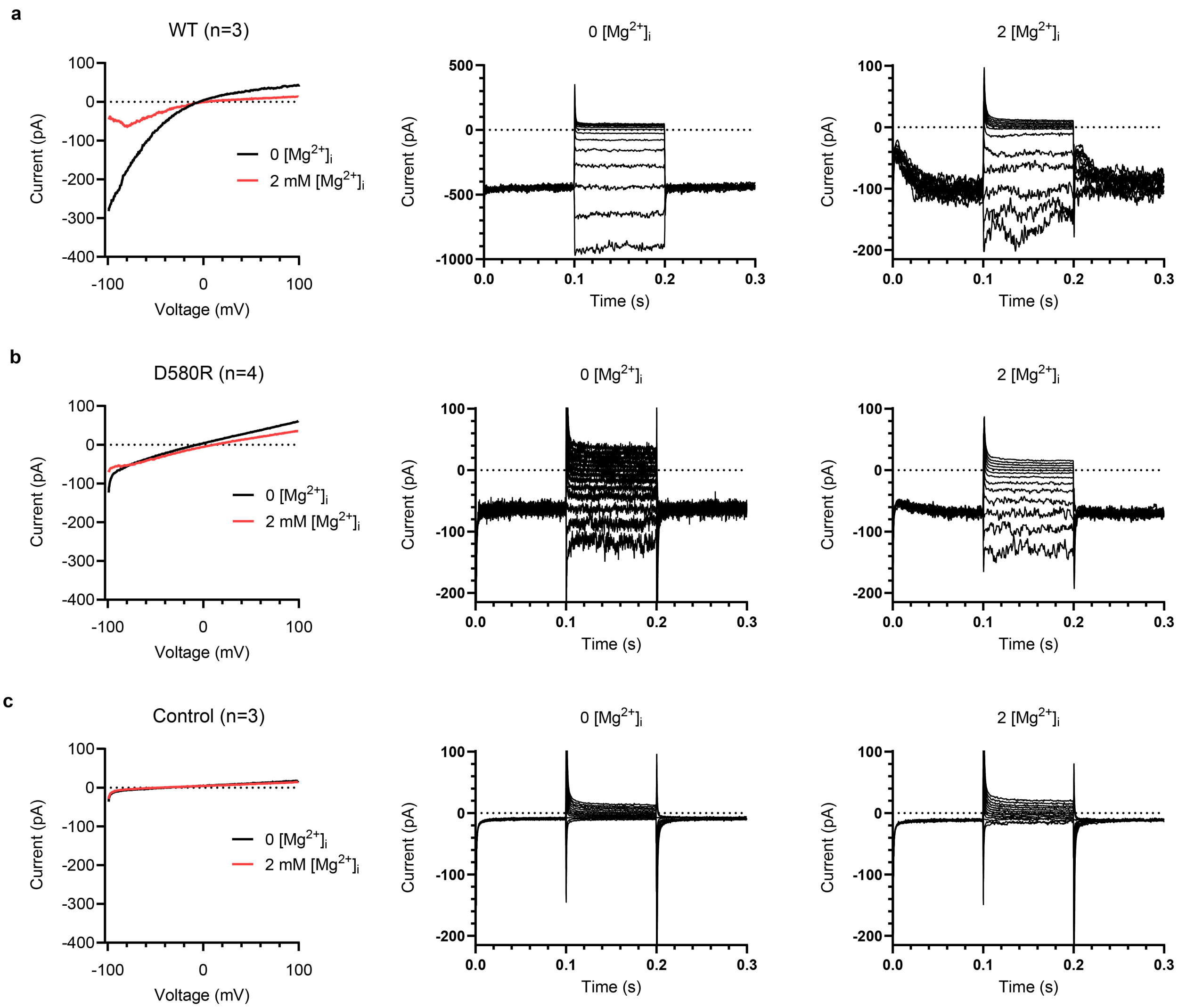

Supplementary Figure 5. Inside-out patch-clamp recordings of WT and D580R hTRPV6-mediated currents in the absence or presence of intracellular Mg^{2+}. a-c, Currents measured from HEK 293 cells transfected with WT hTRPV6 (**a**), hTRPV6-D580R (**b**), or non-transfected (**c**). Left panels show average current amplitudes (n = 3-4 cells) obtained using the ramp protocol illustrated in Fig. 1a. Middle and right panels show representative currents measured in the absence or presence of 2 mM intracellular Mg^{2+} using the voltage step protocol illustrated in Fig. 1c ($n \geq 3$ cells).

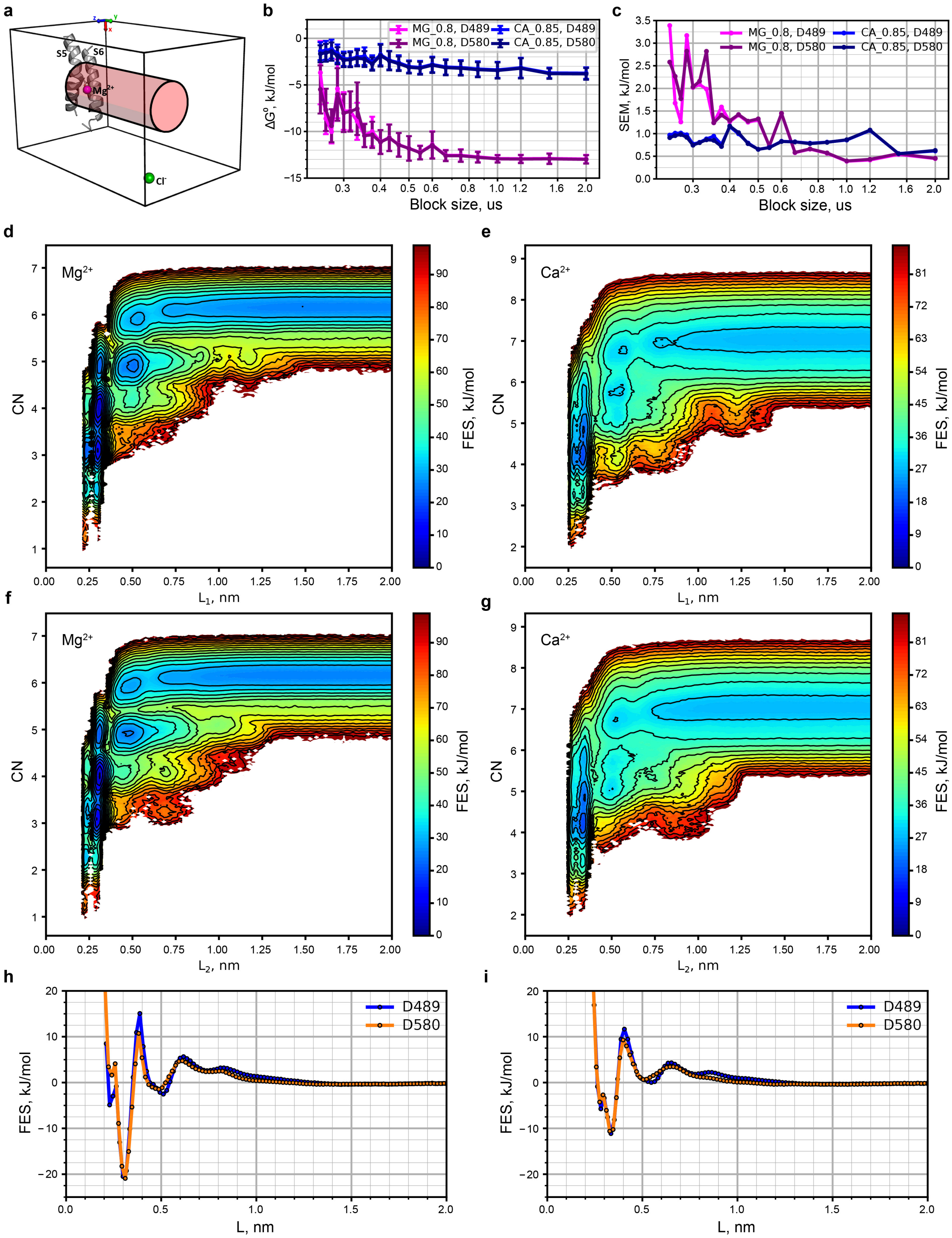

Supplementary Figure 6. Modeling of Mg^{2+} and Ca^{2+} binding to the D489-D580 site in TRPV6$_{Mg}$. a, Schematic representation of the model system for WTMetaD calculations, with the transmembrane helices S5 and S6 shown in cartoon representation, cation as a magenta sphere that can move inside the red cylinder, and Cl^{-} ion as a green sphere. Water molecules are omitted for clarity. **b-c**, Block analysis: the dependencies of mean ΔG^0 values from the block size (**b**), and standard error of the mean (SEM) values from the block size (**c**). **d-g,** 2D projections of FES on L_1-CN_W (**d,e**) and L_2-CN_W (**f,g**) pairs of CVs for Mg^{2+} (**d,f**) and Ca^{2+} (**e,g**). **h-i**, PMFs along the distances from cation to Cγ of D489 (L_1, blue) and D580 (L_2, orange) for Mg^{2+} (**h**) and Ca^{2+} (**i**).

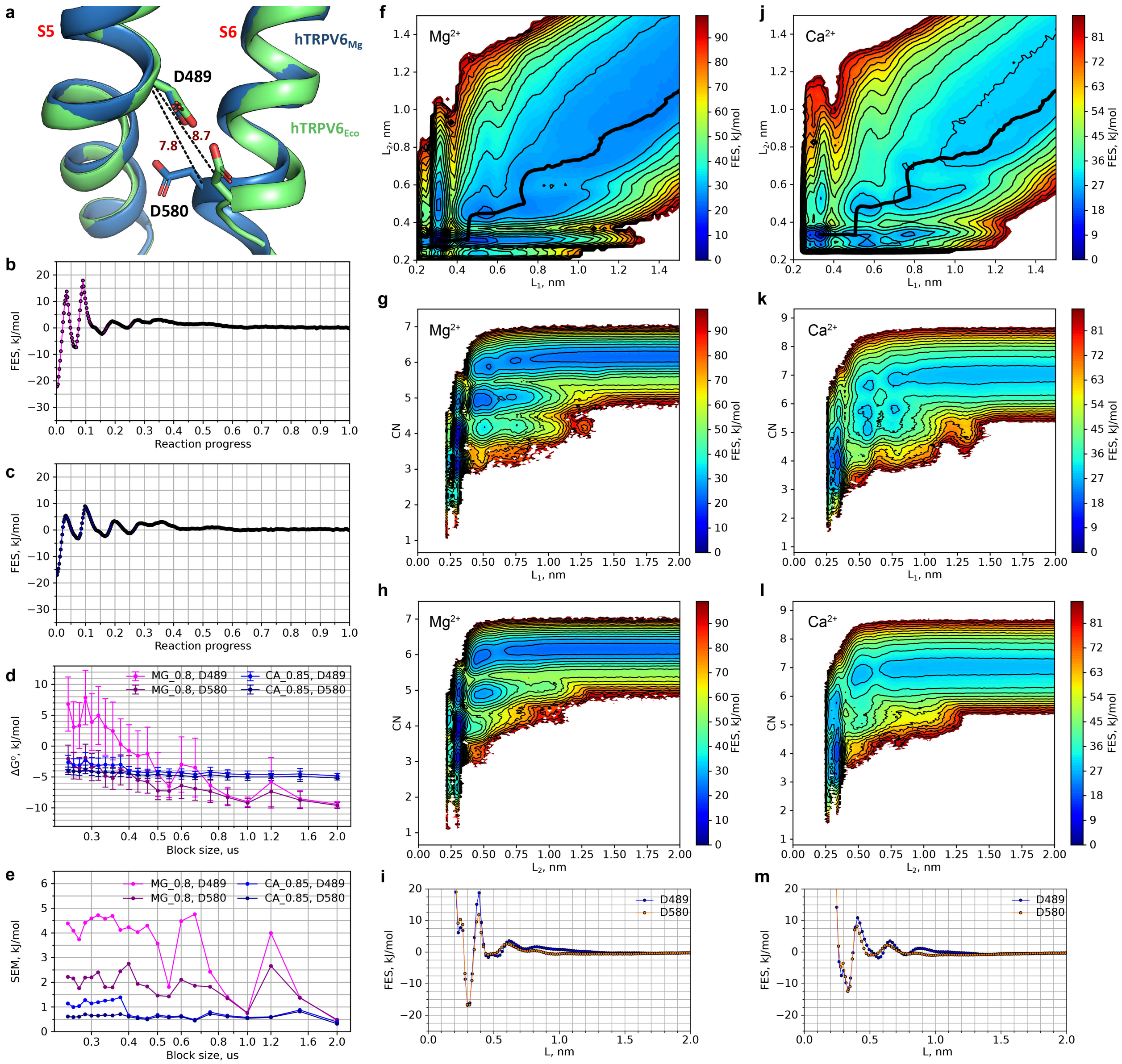

Supplementary Figure 7. Modeling of Mg^{2+} and Ca^{2+} binding to the D489-D580 site of hTRPV6$_{Eco}$. a, D489-D580 site in hTRPV6$_{Mg}$ (blue) and hTRPV6$_{Eco}$ (green). S5 and S6 helices are shown in cartoon representation, D489 and D580 residues are shown as sticks. Distance between Cα atoms of D489 and D580 is shown as dashed lines. **b-c**, Free energy along the MFEPs shown as black lines in panels (**f,j**). **d-e**, Block analysis: the dependencies of mean ΔG^0 values from the block size (**d**), and standard error of the mean (SEM) from the block size (**e**). **f-l**, 2D projections of FES on L_1-L_2 (**f,j**), L_1-CN_W (**g,k**) and L_2-CN_W (**h,l**) pairs of CVs for Mg^{2+} (**f,g,h**) and Ca^{2+} (**j,k,l**). The CVs definition is the same as for TRPV6$_{Mg}$ modeling in Fig. 7 and Supplementary Fig. 6. **i-m**, PMFs along the distances from cation to Cγ of D489 (L1, blue) and D580 (L2, orange) for Mg^{2+} (**i**) and Ca^{2+} (**m**).

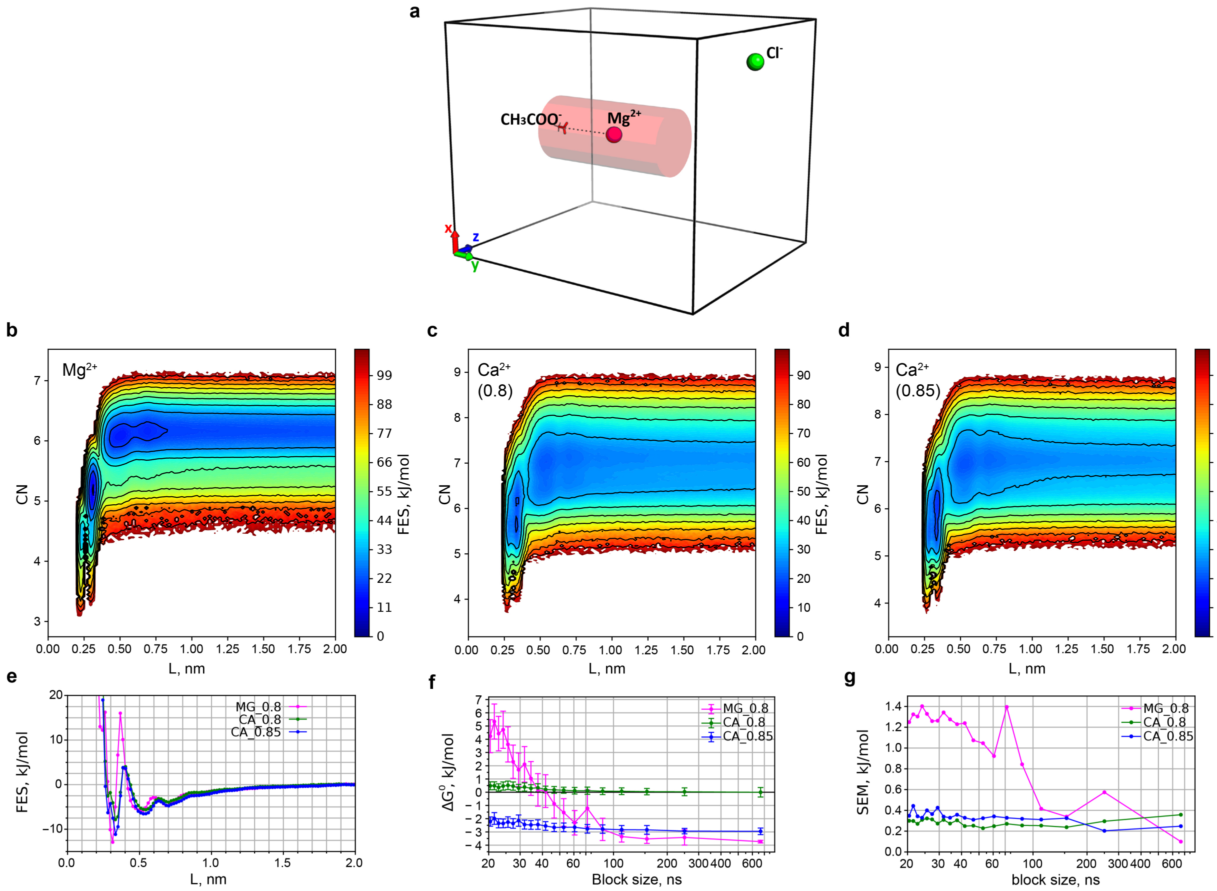

Supplementary Figure 8. Modeling of Mg^{2+} and Ca^{2+} binding to the acetate ion. a, Schematic representation of the model system for WTMetaD simulations, with the acetate ion shown as sticks, cation as a magenta sphere that can move inside the red cylinder, and Cl^{-} ion as a green sphere. Water molecules are omitted for clarity. **b-d**, FES distribution along two CVs, where L is the distance between the cation and carbon atom of the carboxyl group of acetate. CN_W is the cation coordination number of water molecules for Mg^{2+} (**b**), Ca^{2+} with SF = 0.8 (**c**) and Ca^{2+} with SF = 0.85 (**d**). **e**, PMF along L for Mg^{2+} (magenta) and Ca^{2+} (green for SF=0.8, blue for SF=0.85). **f-g**, Block analysis of ΔG^0 errors: the dependence of mean ΔG^0 (**f**) and its SEM (**g**) from the block size, error bars for the ΔG^0 values in **f** are SEM.

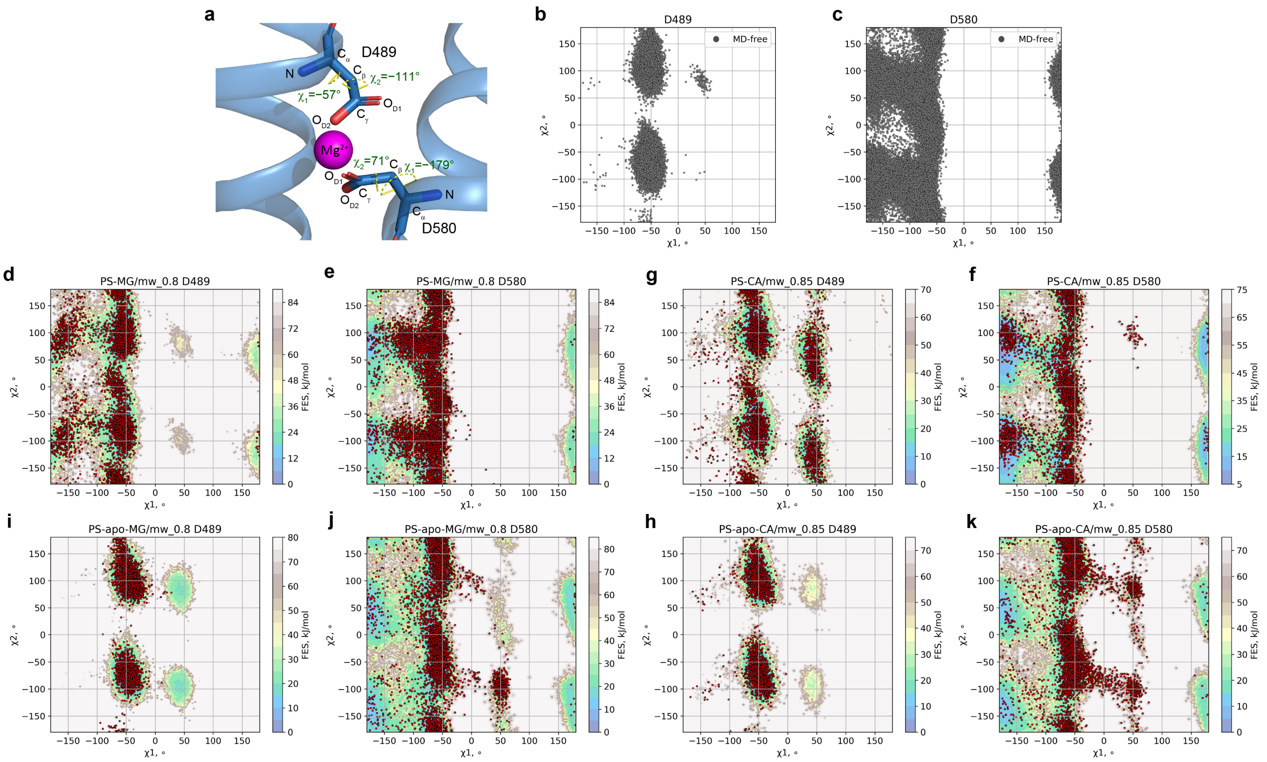

Supplementary Figure 9. Conformational sampling of dihedral angles in PS and PS-apo systems. a, Schematic representation of dihedral angles χ_1 (N-Cα-Cβ-Cγ) and χ_2 (Cα-Cβ-Cγ-O_{D1}); **b-c,** Distribution of χ_1 and χ_2 for D489 (**b**) and D580 (**c**) in unbiased MD simulation. **d-k,** Color maps – WTMetaD free energy surface (FES) projections onto χ_1-χ_2 plane of D489 (**d, g, i, h**) and D580 (**e, f, j, k**) for PS-Mg (**d, e**), PS-Ca (**g, f**), PS-apo-Mg (**i, j**), and PS-apo-Ca (**h, k**). Red dots represent the distribution of “bonded” states (where $L_1 < 0.4$ nm and $L_2 < 0.4$ nm), across the same WTMetaD-trajectories.

Supplementary Table 1. The results of calculations of the standard binding free energy (ΔG^0) and its components for all the modeled systems. SF – charge scaling factor, I – ionic strength, ΔG_{PMF}, ΔG_V, ΔG_I – components of ΔG^0, AS – acetate site, PS – protein site of $TRPV6_{Mg}$, PS-apo – Mg^{2+} free protein site of $TRPV6_{Eco}$. ΔG^0 values are mean ± SEM.

System	SF	I, M	ΔG_{PMF}, kJ/mol	ΔG_V, kJ/mol	ΔG_I, kJ/mol	ΔG^0, kJ/mol	N walkers × simulation time
Mg^{2+}-PS	0.80	0.016	–13.16	0.65	–0.43	–12.9 ± 0.4	4×2 μs
Ca^{2+}-PS	0.85	0.018	–3.86	0.65	–0.52	–3.7 ± 0.6	4×2 μs
Mg^{2+}-PS-apo	0.80	0.016	–9.64	0.65	–0.43	–9.4 ± 0.5	4×2 μs
Ca^{2+}-PS-apo	0.85	0.018	–4.94	0.65	–0.52	–4.8 ± 0.4	4×2 μs
Mg^{2+}-AS	0.80	0.021	–5.17	1.93	–0.50	–3.7 ± 0.1	1×3 μs
Ca^{2+}-AS	0.80	0.021	–1.44	1.93	–0.50	0.0 ± 0.4	1×3 μs
Ca^{2+}-AS	0.85	0.024	–4.29	1.93	–0.60	–3.0 ± 0.2	1×3 μs
